# Zoledronic acid in metastatic chondrosarcoma and advanced sacrum chordoma: two case reports

**DOI:** 10.1186/1756-9966-28-7

**Published:** 2009-01-13

**Authors:** Liliana Montella, Raffaele Addeo, Vincenzo Faiola, Gregorio Cennamo, Rosario Guarrasi, Elena Capasso, Rosanna Mamone, Caraglia Michele, Salvatore Del Prete

**Affiliations:** 1 Medical Oncology Unit "San Giovanni di Dio" Hospital, 80020 Frattaminore, Naples, Italy; 2 Senology Unit, Distretto 65 ASL Napoli3, Arzano, Naples, Italy; 3 Radiology and Diagnostic Imaging Unit, "San Giovanni di Dio" Hospital, Frattaminore, Naples; 4 Department of Biochemistry and Biophysics, Second University of Naples, Italy

## Abstract

**Introduction:**

Chondrosarcomas and chordomas are usually chemoresistant bone tumors and may have a poor prognosis when advanced. They are usually associated with worsening pain difficult to control.

**Patients and Methods:**

Zoledronic acid was used in a 63-year-old man with metastatic chondrosarcoma and in a 66-year-old woman with a diagnosis of sacrum chordoma both reporting severe pain related to tumor.

**Results:**

In the first case, zoledronic acid was able to maintain pain control despite disease progression following chemotherapy, in the other case, zoledronic acid only produced significant clinical benefit.

**Conclusion:**

Control of pain associated with bone tumors such as chondrosarcoma and chondroma may significantly improve from use of zoledronic acid, independently from tumor response to other treatments. Evaluation on larger series are needed to confirm the clinical effect of this bisphosphonate on such tumors.

## Background

Malignant tumors arising from the skeletal system are rare, representing only 0.2% of all new cancers [1]. Bone tumors are classified by cell type and recognized products of proliferating cells. Chondrogenic tumors account for about 21% of bone tumors. Chondrosarcoma is a malignant cartilage forming tumor. Conventional chondrosarcoma is the most frequent type of chondrosarcoma and may develop centrally within the medullary cavity (primary or central chondrosarcoma) or within the cartilage cap of a pre-existing osteochondroma (secondary or peripheral chondrosarcoma). Most chondrosarcomas develop in the thoracic, pelvic and long bones. Grade is the single most important predictive factor for local recurrence and metastasis. Chordoma arises from remnants of notochord and is very rare representing about 3% of bone tumors. Chordomas are characteristically distributed throughout the midline with 50% occurring in the sacrococcygeal region, approximately 35% in the skull base and about 15% in the mobile vertebral column [2]. Both tumors may have a severe prognosis when advanced because of limited curative therapies, poor functional outcome and severe pain. When feasible, aggressive surgery represents the best chance of cure. However, recurrence rate are high. Resistance to chemotherapy makes even more difficult management of sarcoma.

Bisphosphonates are known to inhibit osteoclast-mediated bone resorption and osteoblast differentiation. The evolution of bisphosphonates has led to the development of nitrogen-containing bisphosphonates (N-BPs) which have a different mechanism of action in comparison from that of older nonnitrogen-containing bisphosphonates [3]. N-BPs include pamidronate, alendronate, ibandronate, risedronate and zoledronic acid. Zoledronic acid is the most potent bisphosphonate known to date and has shown to be between 87-fold and 940-fold more potent than pamidronate in animal models of bone resorption [4].

N-BPs exert their effects on osteoclasts and tumor cells by inhibiting a key enzyme in the mevalonate pathway, farnesyl disphosphate synthase, thus preventing protein prenylation and activation of intracellular signalling proteins such as ras [3]. In particular, inhibition of protein prenylation and ras signalling within osteoclasts leads to defects in intracellular vesicle transport. As an example, osteoclasts became defective as concerns ruffled borders which is required for bone resorption.

Bisphosphonates induce caspase-dependent apoptosis, inhibit metalloproteinase activity and have antiangiogenic properties. Reduction in Vascular Endothelial Growth factor (VEGF) levels was showed during pamidronate treatment in cancer patients [5].

The intense effect exerted within bone microenvironment may have a great result not only for metastatic but also for primitive tumors of bone. Recent reports support a direct antitumor activity by zoledronic acid. This effect was documented in cellular and animal models of osteosarcoma [6-8]. Zoledronic acid, paclitaxel alone or associated were tested in a murine model of Ewing sarcoma [8]. Tumor growth was showed in 78% of rats treated with paclitaxel, 44% of rats treated with zoledronic acid and 22% of rats treated with zoledronic acid plus paclitaxel [8]. In this study, paclitaxel and zoledronic acid act synergically despite the minimal antitumor activity of paclitaxel in sarcomas. Therefore the activity of some chemotherapeutic agents may improve in association with zoledronic acid. Many reports are in line with this suggestion [6,8,10]. Preclinical models of chondrosarcoma confirm the effect of zoledronic acid [11]. Insights into molecular mechanisms have demonstrated DNA-damage S-phase checkpoint and up-regulation of mitochondrial permeability independently of p53 and retinoblastoma status [12]. Therefore, zoledronic acid can inhibit cell proliferation and induce apoptosis in tumors where these mutations frequently occur.

Skeletal-related events and bone pain share the same underlying origin. The inhibition of tumor-induced bone resorption by N-BPs produce significant reduction in skeletal morbidity and bone pain [13]. Usually pain is the first symptom of metastatic involvement of bone by tumor. Pain could increase gradually and treatment with opioids or palliative radiation therapy may be required. Typically, bone pain is not adequately managed and 75%–95% of patients with advanced cancer experience severe pain [13]. Treatment with zoledronic acid provides substantial benefit in terms of pain relief in patients with bone metastases by various tumors [14,15].

Zoledronic acid was currently approved worldwide for the treatment of bone metastases independent of the primary tumor type. However, there is no reported clinical experience concerning chondrosarcoma and/or chordoma until now.

Following we report on a 63-year-old man patient with advanced chondrosarcoma and a 66-year-old woman with sacrum chordoma treated with zoledronic acid.

## Case presentation

**Figure 1:**
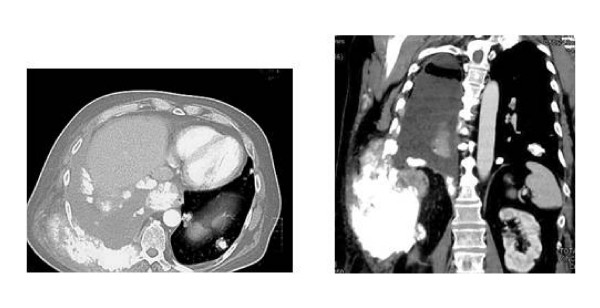
**a Thoracic CT scan in the patient with chondrosarcoma shows at right the lesion involving muscles and ribs**. Lung metastases were visualized. b Coronal section displays the large tumor.

In 2006, a 63-year-old Caucasian man referred a painful swelling at level of right thorax developed during the last month. He was under cardiologic control for mild heart failure. By Computer Tomography (CT) examination a lesion measuring 15 cm maximum diameter involving muscles and ribs was showed. The lesion appeared calcified (fig. 1a and 1b). Concomitant lung metastases, some of them with calcifications, and right pleural effusion were showed (fig. 1a). Bone scintigraphy displayed ligand uptake in the right thorax. Fine needle biopsy revealed spindle cell neoplasm being immunohistochemically positive for vimentin and negative for citokeratin pan and S-100. This tumor was defined as a low grade chondrosarcoma. The patient refused further diagnostic procedures. He reported relentless pain corresponding to the tumor location with increasing need for analgesic drugs. The patient started a chemotherapy regimen based on ifosfamide and uromitexan with monthly zoledronic acid (Zometa; Novartis Pharma, Origgio, Italy) administration. After the first cycle the patient reported a significant benefit on pain and the need for analgesic drugs progressively tapered until stopping. This benefit was confirmed with the following administrations. CT documented stable disease after three months and progression after six cycles. Therefore zoledronic acid was maintained while chemotherapy was stopped. However, pain always remained under control until zoledronic acid was administered, that is for further three months after chemotherapy stopping when the patient died.

**Figure 2:**
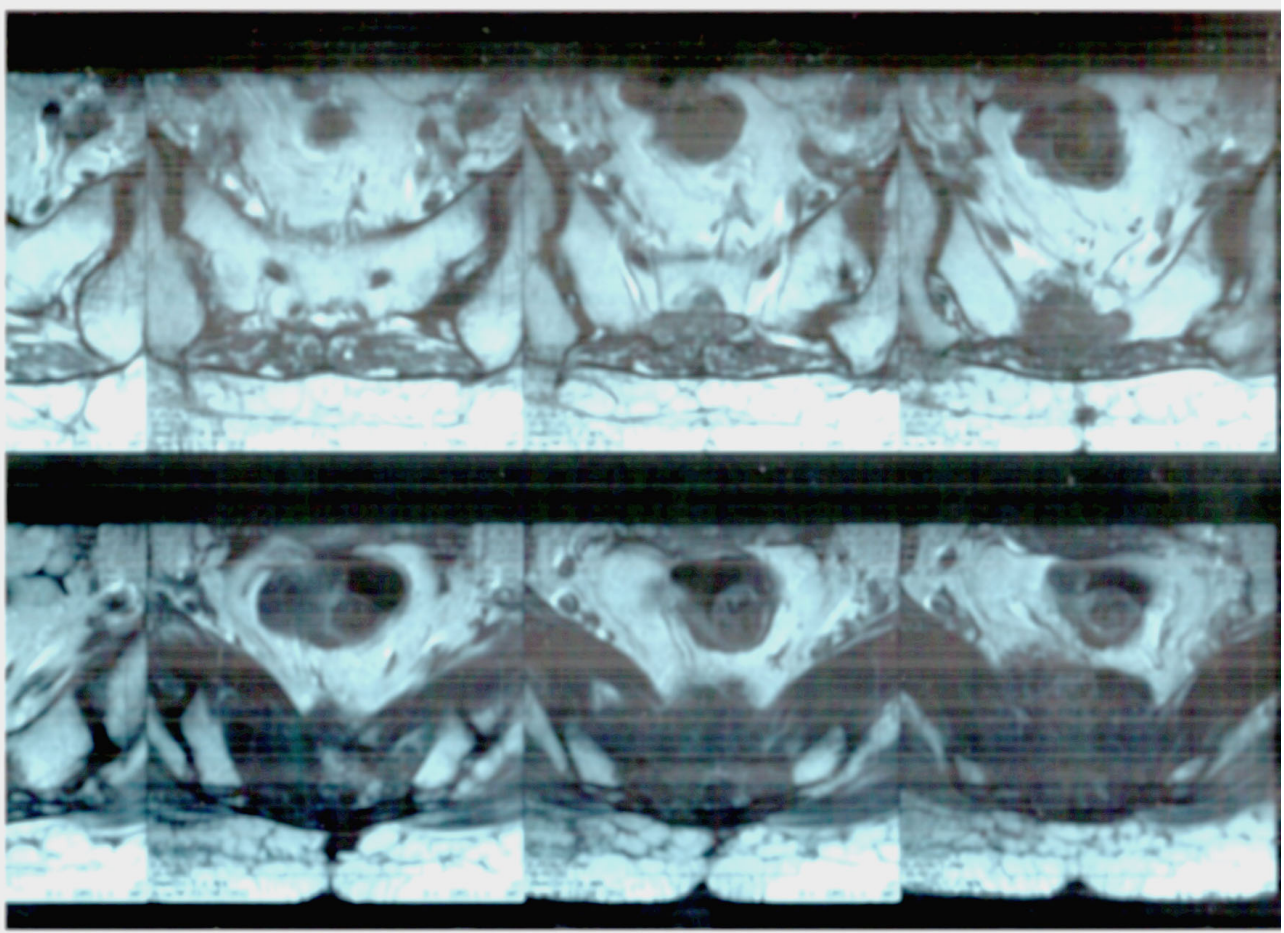
**Pelvic CT scan in the patient with chordoma shows the lesion infiltrating the sacrum**.

In 2002, a 66-year-old Caucasian woman with a history of epilepsy presented progressive lower back pain with irradiation to lower extremities. By sacrum biopsy vacuoled cells having a medium and large size were showed in an abundant myxoid background. These tumor cells were immunohistochemically positive for citokeratin, vimentin and Epithelial Membrane Antigen (EMA) and were weakly positive for S-100. These findings were considered indicative for a sacrum chordoma. The tumor was considered unresectable and treated with radiotherapy. In 2005, despite disease stability by CT scans, the patient complained persisting pain to the sacrum refractory to analgesic, opioids and antiepileptic drugs. Zoledronic acid was started. After few days the patient reported a significant pain reduction. This effect appeared to decrease 20 days after the administration. Therefore, a 21 day-interval of zoledronic acid administration was chosen. The tumor appeared unchanged until now (fig. 2)

## Conclusion

The cases previously reported showed that zoledronic acid may impact on pain related to tumor and quality of life of these patients independently from other administered therapies and disease control. In the patient with chondrosarcoma, chemotherapy and zoledronic acid were concomitantly administrated, therefore the effect of each one cannot be evaluated. However, when the patient progressed, zoledronic acid only was able to maintain pain control.

Zoledronic acid may help in relieving pain related to bone tumors such as chondrosarcoma and chordoma. Studies including more patients are needed to detect the clinical effect of zoledronic acid. However, zoledronic acid appeared to be safe and effective in improvement of pain in the cases described.

## Consent

Written informed consent was obtained by both patients for publication of this report and any accompanying images. A copy of the written consent is available for review by the Editor-in-Chief of this journal.

## Competing interests

The authors declare that they have no competing interests.

## Authors' contributions

All the authors contributed to the acquisition of data, revised the paper and gave final approval.

## References

[B1] Brennan MF, Alektiar KM, Maki RG, De Vita VT, Hellman S, Rosenberg SA (2001). Sarcoma of the soft tissue and bone. Cancer: principles & practice of oncology.

[B2] Tuna H, Aydin V, Bozkurt M, Attar A (2005). Chordoma of the lumbar spine: a case report. Neurocirurgia.

[B3] Green JR (2004). Bisphosphonates: Preclinical review. The Oncologist.

[B4] Green JR, Muller K, Jaeggi KA (1994). Preclinical pharmacology of CGP 42'446, a new, potent, heterocyclic bisphosphonate compound. J Bone Miner Res.

[B5] Santini D, Vincenzi B, Avvisati G, Dicuonzo D, Battistoni F, Gavasci M, Salerno A, Denaro V, Tonini G (2002). Pamidronate Induces Modifications of Circulating Angiogenetic Factors in Cancer Patients. Clin Cancer Res.

[B6] Heymann D, Ory B, Blanchard F, Heymann MF, Coipeau P, Charrier C, Couillaud S, Thiery JP, Gouin F, Redini F (2005). Enhanced tumor regression and tissue repair when zoledronic acid is combined with ifosfamide in rat osteosarcoma. Bone.

[B7] Ory B, Heymann MF, Kamijo A, Gouin F, Heymann D, Redini F (2005). Zoledronic acid suppresses lung metastases and prolongs overall survival of osteosarcoma-bearing mice. Cancer.

[B8] Kubista B, Trieb K, Sevelda F, Toma C, Arrich F, Heffeter P, Elbling L, Sutterlüty H, Scotlandi K, Kotz R, Micksche M, Berger W (2006). Anticancer effects of zoledronic acid against human osteosarcoma cells. J Orthop Res.

[B9] Zhou Z, Guan H, Duan X, Kleinerman ES (2005). Zoledronic acid inhibits primary bone tumor growth in Ewing sarcoma. Cancer.

[B10] Horie N, Murata H, Kimura S, Takeshita H, Sakabe T, Matsui T, Maekawa T, Kubo T, Fushiki S (2007). Combined effects of a third-generation bisphosphonate, zoledronic acid with other anticancer agents against murine osteosarcoma. Br J Cancer.

[B11] Gouin F, Ory B, Redini F, Heymann D (2006). Zoledronic acid slows down rat primary chondrosarcoma development, recurrent tumor progression after intralesional curretage and increases overall survival. Int J Cancer.

[B12] Ory B, Blanchard F, Battaglia S, Gouin F, Rédini F, Heymann D (2007). Zoledronic acid activates the DNA S-phase checkpoint and induces osteosarcoma cell death characterized by apoptosis-inducing factor and endonuclease-G translocation independently of p53 and retinoblastoma status. Mol Pharmacol.

[B13] Lipton A (2007). Treatment of bone metastases and bone pain with bisphosphonates. Support Cancer Ther.

[B14] Kretzschmar A, Wiege T, Al-Batran SE, Hinrichs HF, Kindler M, Steck T, Illiger HJ, Heinemann V, Schmidt K, Haus U, Kirner A, Ehninger G (2007). Rapid and sustained influence of intravenous zoledronic Acid on course of pain and analgesics consumption in patients with cancer with bone metastases: a multicenter open-label study over 1 year. Support Cancer Ther.

[B15] Addeo R, Nocera V, Faiola V, Vincenzi B, Ferraro G, Montella L, Guarrasi R, Rossi E, Cennamo G, Tonini G, Capasso E, Santini D, Caraglia M, Del Prete S (2008). Management of pain in elderly patients receiving infusion of zoledronic acid for bone metastasis: a single-institution report. Support Care Cancer.

